# Editorial: Crosstalk between metabolism and immunity

**DOI:** 10.3389/fmolb.2025.1599219

**Published:** 2025-04-03

**Authors:** Jie Xiong

**Affiliations:** Shanghai Institute of Hematology, State Key Laboratory of Medical Genomics, National Research Center for Translational Medicine at Shanghai, Ruijin Hospital Affiliated to Shanghai Jiao Tong University School of Medicine, Shanghai, China

**Keywords:** metabolism, immunity, infection, oncogenesis, therapeutic targets

Immunometabolism represents an important interdisciplinary field of research, offering novel insights into the pathogenic mechanisms and therapeutic targets of multiple diseases. More recently, the emergence of single-cell, spatial, and *in vivo* metabolomic technologies has now enabled in-depth analysis about the spatiotemporal heterogeneity that is particularly valuable in the field of cancer immunology (Xiao et al., 2024). Besides, during the process of infection, pathogens remodel the metabolism of host to promote their replication and proliferation. Conversely, host cells exploit metabolic mechanism to defend against pathogens (Kreimendahl and Pernas, 2024). In this Research Topic, we are excited to present four outstanding articles discussing recent advances in the regulatory mechanism and functional role of immunometabolism in different diseases, as well as their implications in guiding targeted therapies.

Dyregulating cellular metabolism, avoiding immune destruction and tumor-promoting inflammation are core conceptualization of the hallmarks of cancer (Hanahan, 2022). In this Research Topic, Zhou et al. thoroughly reviewed how lactate metabolism regulating immune function in the disease context of lymphoma. Lymphoma presented with Warburg effect, characterized by high glucose intake, aerobic glycolysis and accumulation of lactate in the tumor microenvironment. Lactic acidosis reduces the infiltration of IFN-γ-producing T and NK cells through inhibiting nuclear factor of activated T cells (NFAT), T cell receptor-triggered JNK/c-Jun and P38 pathways, promotes Treg cells differentiation through the NAD: NADH ratio, induces M2 macrophage polarization though upregulating cyclic adenosine monophosphate (cAMP), early inhibitory protein (ICER), ARG1, VEGF, and HIF-1α, as well as enhances the expression of Programmed cell death-1 (PD-1), Cytotoxic T-lymphocyte-associated protein 4 (CTLA-4) and V-domain ig suppressor of T-cell activation (VISTA). Conversely, metabolism is a complex process regulated by different organs and tissues including immune cells. Implicating in plethora aspect of immune cell development and function, interferon regulatory factors (IRFs) modulate the metabolic pathophysiology through their effects on immune cells and transcriptional actions (Ahmad et al., 2024). Future studies on the regulatory role of immune factors in metabolic pathophysiology as well as the underlying mechanisms are anticipated.

The development of new technologies, such as single-cell transcriptomic sequencing (scRNA-seq), single-nucleus RNA sequencing (snRNA-seq), single-cell assay for transposase-accessible chromatin with high throughput sequencing (ATAC-seq), and cellular indexing of transcriptomes and epitopes sequencing (CITE-seq), etc., provided in-depth understanding of the heterogeneity and cellular interaction in different tissues. Ning et al. focused on the pathologic role of liver non-parenchymal cells, including liver macrophages/Kupffer cells (KCs), liver sinusoidal endothelial cells (LSECs), and hepatic stellate cells (HSCs) in metabolic associated steatohepatitis (MASH), which is the advanced stage of metabolic dysfunction-associated fatty liver disease and can progress to liver fibrosis, cirrhosis and even hepatocellular carcinoma. Monocyte-derived KCs contribute to pro-inflammatory, pro-fibrotic, and immunosuppressive microenvironment, as well as subsequently activate LSECs and HSCs. LSECs further regulate blood flow, facilitate metabolic exchange, modulate local inflammatory responses and immune tolerance. As the driving factor of liver damage and fibrosis, activated HSCs transform into myofibroblast-like cells, secreting excessive extracellular matrix components. Although mouse models with MASH or liver fibrosis share similar pathogenic mechanism with human, sophisticated heterogeneity of macrophages and corresponding marker genes, as well as additional types of pathogenic NPCs were observed. Therefore, better understanding of the inter-kingdom disparities and similarities is important for investigation of molecular mechanisms and therapeutic targets.

Acted as opportunistic pathogens or symbiotic commensals, fungi are associated with inflammatory, infection, metabolic disease, and cancer. Costantini et al. compared the metabolic alterations of tryptophan (Trp), sphingosine-1-phosphate (S1P), and oxalate in human and fungi under different pathological settings. Particularly, the enzymatic repertoire shapes the metabolic crosstalk at human-fungal interface and contributes to immune tolerance or inflammatory pathology, which could be exploited for therapeutic purposes. Mycobiome, predominantly located in oral cavity, skin, lungs, gastrointestinal tract, and urinary tract, is linked to various disease states, such as metabolic inflammatory, neurological, and gastrointestinal disorders (Ratiner et al., 2024). More recently, the presence of the mycobiome has been detected in many cancers, including pancreatic, oral, colorectal, breast, lung, skin, glioblastoma, ovarian and bone cancer, controlling the immune suppression and metabolite composition (Guglietta et al., 2025). Besides, mycobiome assessment has been integrated into data-driven disease risk assessment, diagnosis and treatment, as well as determines treatment responses (Ratiner et al., 2024). However, current mycobiome research mainly focus on their association with cancer progression lacking causative evidence. Development of standard protocols for fungal DNA extraction, purification and subsequent analysis, as well as enrichment of fungal high-throughput sequencing data in multiple sample types derived from different diseases will provide better understanding for the pathophysiological role of fungal.

In addition to microbiota, the circadian clock has emerged as an important factor involving in multiple diseases though regulating immune function and metabolic programming. Luo et al. comprehensively summarized the effect of circadian clock in physiological processes including embryonic development, height and wight gain, and proper activity and function of organs, etc., as well as disease states, including cardiovascular diseases, psychiatric disorders, neurodegenerative diseases, and cancers, etc. Of note, circadian rhythm disturbance induces differentiation and infiltration of anti-inflammatory macrophages and regulatory T cells, as well as inhibits the function of CD8^+^ T cell and dendritic cells, forming an immunosuppressive microenvironment. Besides, the circadian clock also participated in the modulation of various metabolic process, including aerobic glycolysis, *de novo* nucleotide synthesis, glutamine and protein metabolism, lipid metabolism, mitochondrial metabolism, and redox homeostasis, which were oncogenic metabolism fueling tumor cell proliferation (Wang et al., 2024). An improved understanding of how clock components (including circadian clock and clock genes/proteins) regulate the cancer cells metabolism and the tumor microenvironment will inform the development of novel clock-oriented therapeutic strategies.

In conclusion, the contributions to this Research Topic highlighted the fundamental role of metabolism and immunity in both maintaining physiological homeostasis and driving pathological process. Facilitated by cutting-edge technologies, the knowledge about heterogeneity of immune cells and metabolic enzymes inner or inter different species, organs and tissues has remarkably improved, which suggested carefully assessment of the cellular, organoid and animal models for functional study, as well as shed new lights on the molecular mechanisms. Understanding the complex functions of immunometabolism alterations offer potential therapeutic strategies to treat diseases and improve human health.
